# Inhibition of ERK1/2 signaling prevents bone marrow fibrosis by reducing osteopontin plasma levels in a myelofibrosis mouse model

**DOI:** 10.1038/s41375-023-01867-3

**Published:** 2023-03-16

**Authors:** Elisa Bianchi, Sebastiano Rontauroli, Lara Tavernari, Margherita Mirabile, Francesca Pedrazzi, Elena Genovese, Stefano Sartini, Massimiliano Dall’Ora, Giulia Grisendi, Luca Fabbiani, Monica Maccaferri, Chiara Carretta, Sandra Parenti, Sebastian Fantini, Niccolò Bartalucci, Laura Calabresi, Manjola Balliu, Paola Guglielmelli, Leonardo Potenza, Enrico Tagliafico, Lorena Losi, Massimo Dominici, Mario Luppi, Alessandro Maria Vannucchi, Rossella Manfredini

**Affiliations:** 1 Centre for Regenerative Medicine “Stefano Ferrari”, University of Modena and Reggio Emilia, Modena, Italy; 2 Department of Life Sciences, University of Modena and Reggio Emilia, Modena, Italy; 3 Department of Biomedical, Metabolic and Neural Sciences, University of Modena and Reggio Emilia, Modena, Italy; 4 Evotec (Modena) Srl, Medolla, MO Italy; 5 Division of Oncology, Laboratory of Cellular Therapy, Department of Medical and Surgical Sciences of Children & Adults, University of Modena and Reggio Emilia, Modena, Italy; 6 Department of Medical and Surgical Sciences of Children & Adults, Pathology Unit, University of Modena and Reggio Emilia, Modena, Italy; 7 Department of Laboratory Medicine and Pathology, Diagnostic Hematology and Clinical Genomics, AUSL/AOU Policlinico, 41124 Modena, Italy; 8 Center Research and Innovation of Myeloproliferative Neoplasms (CRIMM), Department of Experimental and Clinical Medicine, AOU Careggi, University of Florence, Florence, Italy; 9 Department of Medical and Surgical Sciences, University of Modena and Reggio Emilia, AUSL/AOU Policlinico, 41124 Modena, Italy; 10 Department of Life Sciences, Pathology Unit, University of Modena and Reggio Emilia, Modena, Italy

**Keywords:** Myeloproliferative disease, Cell signalling, Pathogenesis

## Abstract

Clonal myeloproliferation and development of bone marrow (BM) fibrosis are the major pathogenetic events in myelofibrosis (MF). The identification of novel antifibrotic strategies is of utmost importance since the effectiveness of current therapies in reverting BM fibrosis is debated. We previously demonstrated that osteopontin (OPN) has a profibrotic role in MF by promoting mesenchymal stromal cells proliferation and collagen production. Moreover, increased plasma OPN correlated with higher BM fibrosis grade and inferior overall survival in MF patients. To understand whether OPN is a druggable target in MF, we assessed putative inhibitors of OPN expression in vitro and identified ERK1/2 as a major regulator of OPN production. Increased OPN plasma levels were associated with BM fibrosis development in the Romiplostim-induced MF mouse model. Moreover, ERK1/2 inhibition led to a remarkable reduction of OPN production and BM fibrosis in Romiplostim-treated mice. Strikingly, the antifibrotic effect of ERK1/2 inhibition can be mainly ascribed to the reduced OPN production since it could be recapitulated through the administration of anti-OPN neutralizing antibody. Our results demonstrate that OPN is a novel druggable target in MF and pave the way to antifibrotic therapies based on the inhibition of ERK1/2-driven OPN production or the neutralization of OPN activity.

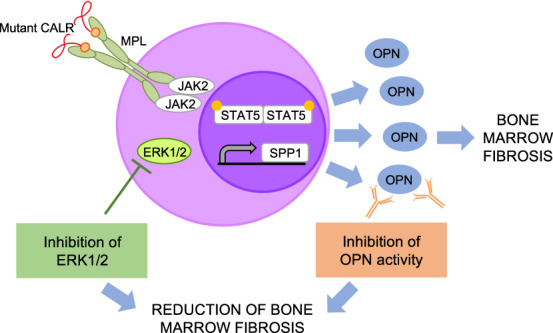

## Introduction

Philadelphia-negative myeloproliferative neoplasms (MPNs) are characterized by clonal myeloproliferation. Among them, myelofibrosis (MF) is characterized by the development of bone marrow (BM) fibrosis [1], together with extramedullary hematopoiesis, constitutional symptoms, leukemic transformation, and shortened life expectancy. The hyperproliferation of the neoplastic clone and the overproduction of proinflammatory and profibrotic mediators are the two major events that drive the onset and progression of MF.

Great efforts aimed to unveil the molecular landscape of MPNs have led to the identification of the genomic lesions responsible for the clonal myeloproliferation, primarily the “driver” mutations affecting *JAK2*, *MPL,* and *CALR* genes that converge towards the constitutive activation of JAK/STAT signaling [2–5]. Several mechanisms underlying the abnormal production of proinflammatory and profibrotic mediators, such as reactive oxygen species [6, 7] and interleukins (e.g., IL-6) [8, 9], and the skewed crosstalk between the malignant hematopoietic cells and BM microenvironment, have been recently unveiled. Nevertheless, the elucidation of mechanisms leading to the development of BM fibrosis is still of primary importance to develop novel therapeutic strategies aimed to reverse the alteration of BM architecture and function. Current therapeutic options effectively improve splenomegaly, constitutional symptoms and may improve anemia but do not efficiently reverse BM fibrosis [10–12]. Among the experimental therapies, Recombinant interferon-α (rIFN-α) has proven to normalize BM architecture and determine a clinical benefit in a sizable fraction of early stage PMF patients [13, 14]. In addition, the treatment with rIFN-α has been recently associated with a lower risk of MF progression and a lower mortality in comparison with hydroxyurea and phlebotomy in PV patients [15]. However, randomized trials are required to consolidate the impact of rIFN-α therapy on BM hystopathology. The development of novel therapeutic strategies to counteract the skewing of the BM architecture in addition to the clonal myeloproliferation is therefore one of the major unmet needs in MF management.

We previously demonstrated that the upregulation of the transcription factor MAF prompts the massive release of proinflammatory and profibrotic cytokines and growth factors in differentiated myeloid cells (mainly monocytes and megakaryocytes) derived from the malignant CD34 + HPCs. We focused on *SPP1*, the gene coding for osteopontin (OPN), and showed that *SPP1* is a transcriptional target of MAF and that the overproduction of OPN at least partially contributes to the increased fibroblast proliferation and collagen production that underlies the development of myelofibrosis. OPN plasma levels resulted significantly increased in PMF patients in comparison with healthy donors and were remarkably higher in overt fibrotic versus pre-fibrotic PMF patients. Higher OPN plasma levels also correlated with shorter overall survival. Collectively our data point to a role of OPN in the pathogenesis of myelofibrosis, and especially in the development of BM fibrosis [16].

*JAK2*, *MPL* and *CALR* mutations converge toward the STAT5-mediated [2, 17, 18] induction of MAF expression [19] and in turn MAF transactivates the expression of SPP1. JAK1/2 inhibition by Ruxolitinib indeed reduced SPP1 expression in PMF HPCs and monocytes [16]. However, other mechanisms are likely involved to explain the overproduction of OPN since no remarkable differences in OPN plasma levels were found among *JAK2*, *CALR*-mutated and triple-negative PMF patients [16].

Here, we unveil that ERK1/2 signaling contributes to support the production of OPN since treatment with ERK1/2 inhibitor suppresses OPN secretion in vitro and in vivo. We show that pharmacological inhibition of OPN production by the administration of the ERK1/2 inhibitor Ulixertinib negatively interferes with the development of BM and spleen fibrosis in a MF mouse model. We also demonstrate that the antifibrotic effect of ERK1/2 inhibition can be mainly ascribed to the reduction of OPN expression and function, since the inhibition of OPN function through the administration of anti-OPN neutralizing antibody was able to constrain the development of BM and spleen fibrosis in a similar way to ERK1/2 inhibition.

## Methods

### Ethic statements

Human CD14+ monocytes were purified upon donor’s informed written consent from peripheral blood samples. This study was conducted in accordance with the Declaration of Helsinki and after the approval by local ethics committees.

### Compounds and reagents

Ulixertinib (BVD-523, cat# HY-15816) and Ruxolitinib (INCB18424, cat# HY-50856) were purchased by MedChemExpress (Monmouth Junction, NJ, USA).

The thrombopoietin mimetic Romiplostim (Nplate^®^) was kindly provided by Amgen (Thousand Oaks, CA, USA).

Anti-mouse OPN antibody (cat#BE0373, clone 103D6), the isotype control antibody (cat#BE0366, clone DV5-1) and the dilution buffer (cat#IP0070) were purchased by Bio X Cell (Lebanon, NH, USA).

### Mice

All animal studies were reviewed and approved by the Italian Ministry of Health (approval 23/2016_PR). Wild-type C57BL/6J mice were purchased from Charles River Laboratories (Wilmington, MA, USA). Mice were housed at the SPF animal facility of the University of Modena and Reggio Emilia, Modena, Italy. The Romiplostim-induced MF mouse model was established through the subcutaneous injection of either vehicle (saline) or Romiplostim (1 mg/kg of body weight; Amgen) once a week for 2 weeks in 6-to-8 weeks-old wild-type C57BL/6J mice, according to Maekawa et al. [20].

Ulixertinib 100 mg/kg, Ruxolitinib 60 mg/kg, a combination of Ulixertinib 75 mg/kg and Ruxolitinib 30 mg/kg or an equal volume of vehicle (i.e., DMSO 10% (v/v) in SBE-β-CD 20% (w/v) in saline) were orally administered by gavage twice daily.

Anti-mouse OPN antibody (15 mg/kg of body weight) or the isotype control antibody (15 mg/kg) were intraperitoneally injected once every 3 days for 2 weeks. Doses and administration schedule were selected based on previous studies [21, 22]. Blood samples were collected in K2-EDTA tubes (BD microtainer, cat#365974, BD, Franklin Lakes, NJ, USA) by submandibular vein puncture and analyzed for hematological parameters at baseline (day 1, i.e., the day of the first administration of Romiplostim) and at days 4, 8, 11, and 15 by a Heska HT5 hematology analyzer (Heska, Loveland, CO, USA). Plasma samples were collected from all the blood samples. Spleen size was evaluated by ultrasound (US) Vevo 2100 Imaging System (FUJIFILM VisualSonics Inc., Bothell, WA, USA). Then, spleen volume was quantified using Vevo Lab 3.1.0 software (FUJIFILM VisualSonics Inc.). In detail, each spleen was divided in multiple sections by software and the drawing of spleen area in each slide allowed the software to automatically calculate spleen volume.

Mice were killed at day 15. The spleen index (i.e., spleen weigh/body weigh x100) was calculated. Spleens and femurs were fixed for 6 h in 10% buffered formalin (cat#05-01V15PKF, Bio-Optica Milan, Italy) for histological analyses.

Quantitation of BM fibrosis was performed by a pathologist in keeping with the WHO grading criteria [23] integrated with the addition of intermediate levels of fibrosis grading (MF-0.5 and MF-1.5) [24–26] as detailed in Table 1.

**Table 1:** Criteria for bone marrow fibrosis grading.

Grading	Description
MF-0	no reticulin fibers corresponding to normal bone marrow
MF-0.5	focal areas with a loose network of reticulin with many intersections alternated with areas with no reticulin fibers
MF-1	loose network of reticulin with many intersections evenly distributed over the entire length of the femurs
MF-1.5	focal areas with a diffuse and dense increase in reticulin with extensive intersections
MF-2	diffuse and dense increase in reticulin with extensive intersections evenly distributed over the entire length of the femurs, occasionally with only focal bundles of collagen and/or focal osteosclerosis
MF-3	diffuse and dense increase in reticulin with extensive intersections with coarse bundles of collagen, often associated with significant osteosclerosis

Plasma samples, BM, and spleen sections of JAK2^floxed/+^ and JAK2^V617F/+^ mice [27] were provided by A.M. Vannucchi.

### Statistical analysis

Values are reported as mean and standard deviation for normally distributed data, or median and interquartile range for non-normally distributed values. The one-way ANOVA was performed to compare normally distributed values while the rank-based nonparametric Kruskal-Wallis test was performed for data with a nonparametric distribution. *P* values were considered statistically significant when <0.05. Statistical analyses were performed by using GraphPad Prism version 9 (GraphPad Software, San Diego, CA, USA).

## Results

### ERK1/2 inhibition reduces OPN production in vitro

**Fig. 1:**
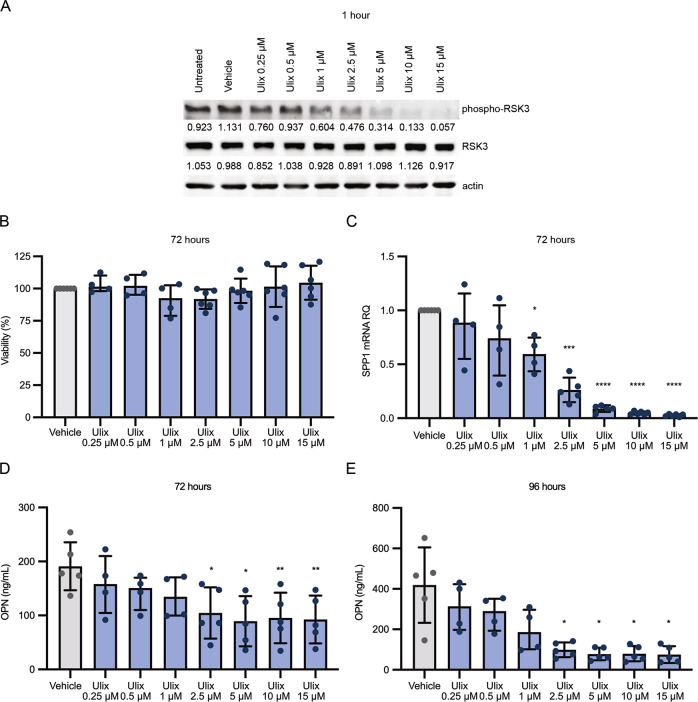
ERK1/2 inhibition using Ulixertinib reduces OPN production by monocytes in vitro. Normal human monocytes were treated with increasing concentration of ERK1/2 inhibitor Ulixertinib. **A** Western blot analysis of phospho-RSK3 protein levels in monocyte after Ulixertinib treatment. **B** Monocytes viability was evaluated using XTT assay after 72 h of treatment. **C** *SPP1* mRNA expression was evaluated by means of real time qRT-PCR after 72 h of treatment. OPN production by monocytes was assessed by means of ELISA (Enzyme-linked immunosorbent assay) in culture supernatants at 72 (**D**) and 96 hours (**E**) of treatment. Histograms represent the mean values while bars indicate the standard deviation. Comparisons were performed by means of one-way ANOVA. *: *P* ≤ 0.05; **: *P* ≤ 0.01; ***: *P* ≤ 0.001; ****: *P* ≤ 0.0001 Abbreviations: SPP1 osteopontin; RQ relative quantity; OPN osteopontin; Ulix Ulixertinib.

Inhibitors of signaling pathways reported in literature to affect OPN expression [28–31] were evaluated in vitro for their ability to reduce OPN mRNA levels. Drugs clinically approved or currently tested in clinical trials and therefore endowed with a translational potential were selected and assayed in vitro in human primary CD14 + monocytes, that are one of the major sources of OPN among the neoplastic clone-derived hematopoietic cells in MF patients [16]. Among them, we focused on Ulixertinib given its ability to inhibit OPN expression (Fig. 1), unlike the other drugs tested (Supplementary Table 1).

Ulixertinib was selected as a potent and selective ERK1/2 inhibitor in order to counteract MAPK signaling pathway that has been reported to promote OPN production [30]. The remarkable reduction of phosphorylated RSK3 levels in treated monocytes demonstrated that Ulixertinib efficiently inhibited ERK1/2 activity in vitro (Fig. 1A). Increasing concentrations of Ulixertinib (0.25-to-15μM) did not affect cell viability (Fig. 1B) while remarkably reduced *SPP1* expression levels detected by Real Time qRT-PCR (Fig. 1C) and OPN secretion evaluated by ELISA on culture supernatants (Fig. 1D, E) in human primary monocytes.

### OPN plasma levels are increased in mice developing BM fibrosis

**Fig. 2:**
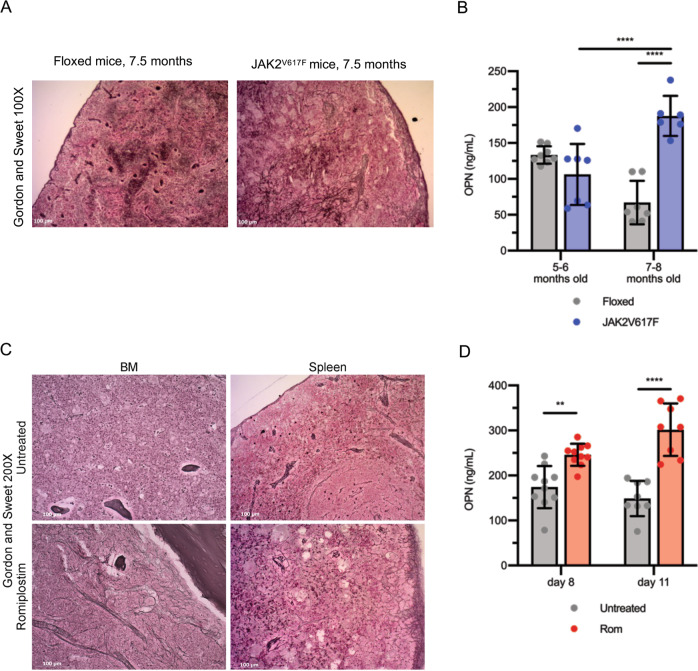
Plasma OPN is increased in MF mouse models. **A** Representative images of Gordon and Sweet’s reticulin staining of spleen sections from JAK2^V617F/+^ mice and control JAK2^floxed/+^ animals 7.5 months after birth. Magnification 100×. **B** OPN plasma levels were evaluated by means of ELISA (enzyme-linked immunosorbent assay) in JAK2^V617F/+^ and JAK2^floxed/+^ mice at 5-6 months and 7–8 months after birth. (*n* = 6–8/group). **C** BM and spleen fibrosis detection by Gordon and Sweet’s reticulin staining in mice treated with thrombopoietin mimetic Romiplostim (Rom) and control animals (Untreated). Magnification 200×. **D** Protein levels of OPN were assessed by means of ELISA in plasma from mice treated with Romiplostim or in control animals 8 and 11 days after the first administration (*n* = 8–10/group). Histograms represent mean values while bars indicate the standard deviation. Comparisons were performed by means of one-way ANOVA. *: *P* ≤ 0.05; **: *P* ≤ 0.01; ***: *P* ≤ 0.001; ****: *P* ≤ 0.0001 Abbreviations: OPN osteopontin; Rom romiplostim; BM bone marrow.

Next, we moved on to study the in vivo effects of OPN reduction in the development of BM fibrosis. To identify the most suitable mouse model, OPN plasma levels were firstly assessed in JAK2^V617F/+^ mice [27], that show a Polycythemia Vera-like phenotype and develop BM and spleen fibrosis with a late onset (i.e., from 7 months of age, Fig. 2A). Noteworthy, OPN plasma levels were remarkably increased in 7-to-8 months-old JAK2^V617F/+^ mice in comparison with age-matched JAK2^floxed/+^ and 5-to-6 months-old JAK2^V617F/+^ mice (Fig. 2B). However, in order to identify a model that develops BM fibrosis in a shorter time we took advantage of the MF mouse model induced by the administration of the thrombopoietin mimetic Romiplostim [20] that develops an overt BM and spleen fibrosis after 15 days of treatment (Fig. 2C). As shown in Fig. 2D, the development of BM and spleen fibrosis was associated with a remarkable increase of OPN plasma levels also in Romiplostim-treated mice. Given the dosing scheme of Ulixertinib (per os twice daily) and due to the potential adverse effects of a frequent and prolonged administration by gavage in mice, we focused on the Romiplostim-treated mice as the best model to evaluate the effects of OPN reduction on the development of BM and spleen fibrosis.

### Ulixertinib costrains OPN production and BM fibrosis in MF mouse model

**Fig. 3:**
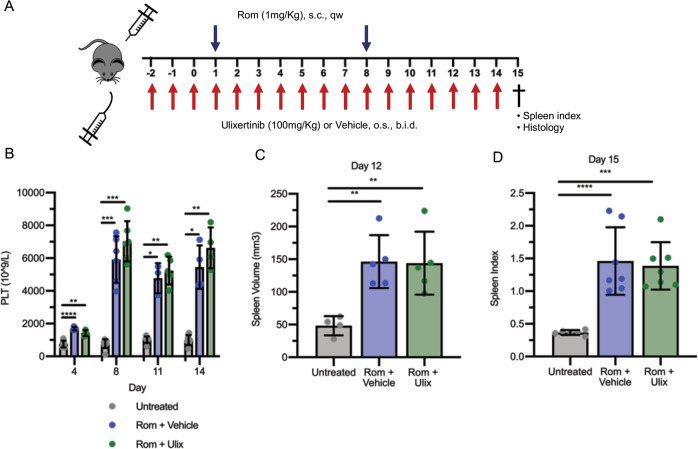
ERK1/2 inhibition does not affect thrombocytosis and splenomegaly induced by Romiplostim. **A** Schematic outline of the experimental design. Mice were given Romiplostim 1 mg/kg through sub-cutaneous injection once weekly. ERK1/2 inhibitor Ulixertinib 100 mg/kg or vehicle (10% DMSO in 20% SBE-β-CD in saline) was administered through oral gavage twice daily starting 3 days before the first Romiplostim injection. Mice were sacrificed after 15 days of Romiplostim treatment. **B** Platelet count of control mice (Untreated, in gray, *n* = 6–9/group), mice treated with Romiplostim alone (Rom + Vehicle, in blue, *n* = 4–6/group) and animals treated with Romiplostim and Ulixertinib (Rom + Ulix, in green, *n* = 4–6/group). Platelet count was assessed at days 4, 8, 11, and 14. Spleen volume (**C**) was assessed by means of Vevo2100 ultra sound system at day 12 (*n* = 4–5/group) while spleen index (**D**) was calculated at sacrifice (day 15) (*n* = 6–7/group). Histograms represent mean values while bars indicate the standard deviation. Comparisons were performed by means of one-way ANOVA. *: *P* ≤ 0.05; **: *P* ≤ 0.01 ***: *P* ≤ 0.001; ****: *P* ≤ 0.0001 vs Untreated. Abbreviations: s.c. sub-cutaneous; qw once weekly; o.s. oral gavage; b.i.d. twice daily; PLT platelets; Rom Romiplostim; Ulix ulixertinib.

We therefore focused on the effects of ERK1/2 inhibition mediated by Ulixertinib in Romiplostim-treated mice. Ulixertinib was dosed twice daily from 3 days before the first administration of Romiplostim (set as day 1) and until the sacrifice (day 15, Fig. 3A). Blood count analysis showed the expected Romiplostim-induced severe thrombocytosis that was not reversed by Ulixertinib (Fig. 3B). Other hematological parameters such as mean platelet volume (MPV), white blood cell (WBC), red blood cell (RBC), hemoglobin (Hb) and hematocrit (HCT) were similarly unaffected by ERK1/2 inhibition (Fig. S1). In addition, the treatment with Ulixertinib did not prevent the development of splenomegaly, that was evaluated in vivo at day 12 by Vevo 2100 ultrasound quantitative analysis of the spleen volume (Figs. 3C and S2) and after sacrifice at day 15 (data reported as spleen index, Fig. 3D). Noteworthy, we observed a strong correlation (*R*^2^ = 0.8674) between the spleen volume evaluated by ultrasound imaging and spleen index calculated post-mortem, at the same time point (Fig. S2E) demonstrating that the ultrasound evaluation of the spleen volume can provide a quantification of splenomegaly and reliable as the spleen index without requiring sacrifice.

**Fig. 4:**
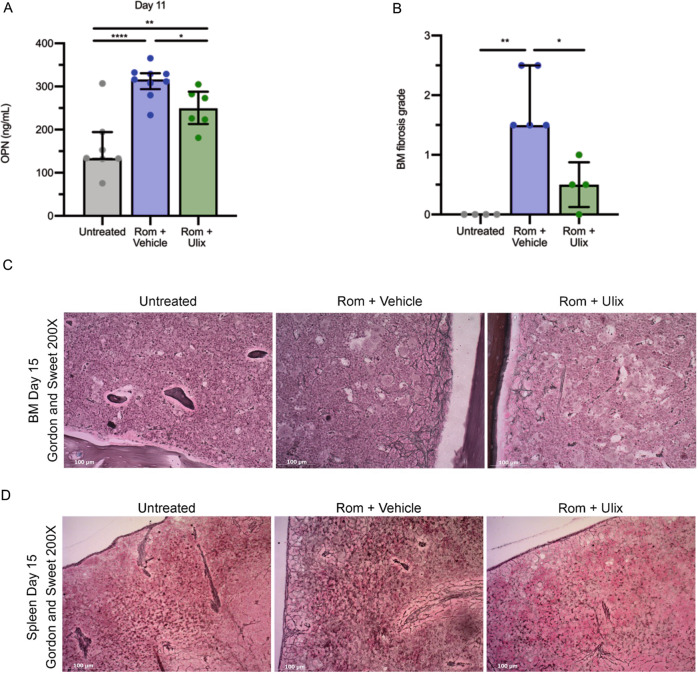
ERK1/2 inhibition reduces OPN plasma concentration and reverses BM fibrosis induced by Romiplostim. **A** OPN plasma concentration was evaluated by means of ELISA (enzyme-linked immunosorbent assay) in control mice (Untreated) and Romiplostim-treated animals receiving vehicle (Rom + Vehicle, in blue) or Ulixertinib (Rom + Ulix, in green). ELISA was performed 11 days after the first Romiplostim administration (*n* = 6–9/group). Histograms represent mean values while bars indicate the standard deviation. Comparisons were performed by means of one-way ANOVA. **B** Blinded fibrosis grade quantification was performed by a specialized pathologist. Histograms represent median values while bars indicate the interquartile range (*n* = 4-5/group). Comparisons were performed by means of Kruskal-Wallis’ test. **C** Representative images of Gordon and Sweet’s reticulin staining of bone marrow (BM) sections from Untreated, Rom + Vehicle and Rom + Ulix mice. Magnification 200×. **D** Spleen fibrosis as shown by Gordon and Sweet’s reticulin staining. Magnification 200×. *: *P* ≤ 0.05; **: *P* ≤ 0.01; ***: *P* ≤ 0.001; ****: *P* ≤ 0.0001 Abbreviations: OPN osteopontin; Rom romiplostim; Ulix Ulixertinib; BM bone marrow.

Conversely, ERK1/2 inhibition had a major effect on OPN production and BM fibrosis. The increase of OPN production in Romiplostim-treated mice was significantly constrained by the administration of Ulixertinib, as demonstrated through the quantification of OPN plasma levels at 11 days (Fig. 4A). In addition, lower OPN plasma levels in Ulixertinib-treated mice were associated with a remarkable reduction of reticulin deposition in BM (Fig. 4B, C) and spleen (Fig. 4D). The histological examination upon Gordon and Sweet silver staining and the fibrosis grading of BM sections (Fig. 4B, C) highlighted the extensive deposition of coarse reticulin fibers with multiple intersections over the entire length of the femurs in MF mice receiving the vehicle. On the contrary, MF mice treated with Ulixertinib developed a remarkably lower level of BM fibrosis characterized by areas with no detectable reticulin fibers alternated with areas showing a loose network of thin reticulin fibers and very few foci with intersections of more dense fibers.

Spleen fibrosis was similarly reduced in MF-mice dosed with Ulixertinib in comparison with those receiving the vehicle (Fig. 4D).

### The combination of Ulixertinib and Ruxolitinib reduces OPN plasma levels and BM fibrosis in MF mice

**Fig. 5:**
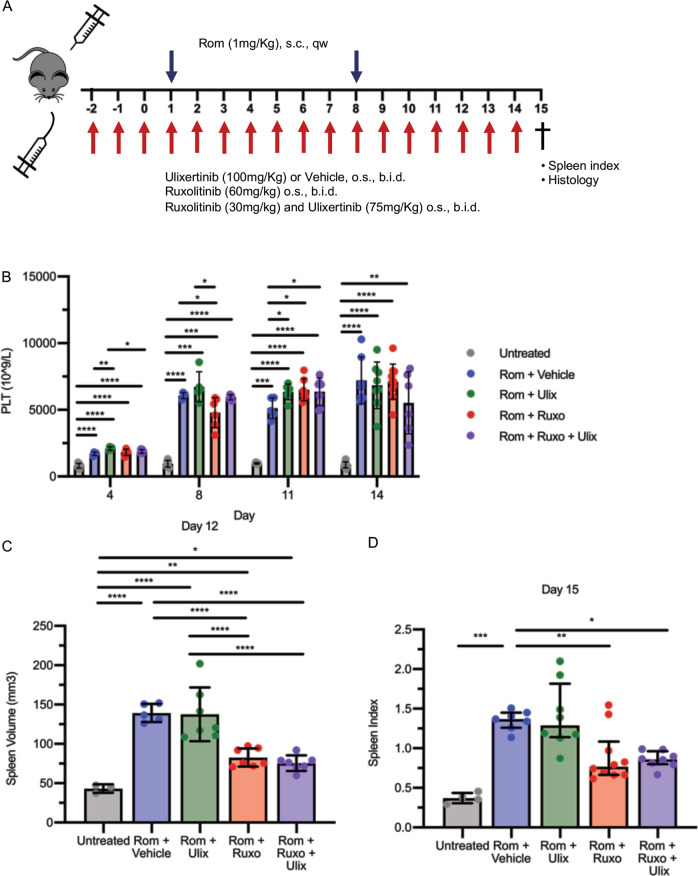
Combined ERK1/2 and JAK1/2 inhibition does not affect thrombocytosis but improves splenomegaly induced by Romiplostim. **A** Schematic outline of the experimental design. Myelofibrosis was induced by sub-cutaneous injection of Romiplostim (1 mg/kg, once weekly). Mice were given vehicle (10% DMSO in 20% SBE-β-CD in saline) (Rom + Vehicle, in blue), Ulixertinib 100 mg/kg (Rom + Ulix, in green), Ruxolitinib 60 mg/kg (Rom + Ruxo, in red) or a combination of Ulixertinib 75 mg/kg and Ruxolitinib 30 mg/kg (Rom + Ruxo + Ulix, in purple) through oral gavage twice daily starting 3 days before the first Romiplostim injection. Mice were sacrificed after 15 days of Romiplostim treatment. **B** Platelet count was assessed at days 4, 8, 11, and 14 (*n* = 4–10/group). Spleen volume (**C**) was assessed by means of Vevo2100 ultra sound system at day 12 (*n* = 3-7/group) while spleen index (**D**) was calculated at sacrifice (day 15) (*n* = 4–10/group). In Panels B and C histograms represent mean values while bars indicate the standard deviation. Comparisons were performed by means of one-way ANOVA. Histograms in panel D represent median values while bars indicate the interquartile range. Comparisons were performed by means of Kruskal-Wallis’ test. *: *P* ≤ 0.05; **: *P* ≤ 0.01. Abbreviations: s.c. sub-cutaneous; qw once weekly; o.s. oral gavage; b.i.d. twice daily; PLT platelets; Rom Romiplostim; Ulix ulixertinib, Ruxo Ruxolitinib.

To date, JAK1/2 inhibition provides a therapeutic benefit displaying only limited effects on BM fibrosis both in patients and mouse models [10–12]. To assess whether the anti-fibrotic activity of Ulixertinib is maintained when coupled with JAK1/2 inhibitors, we compared the effects of the combination of Ulixertinib and Ruxolitinib with those of the treatment with single agents in MF mice (Fig. 5A).

None of the conditions tested was able to counteract the increase of platelet count (Fig. 5B) and volume (MPV, Fig. S3A) induced by Romiplostim. In a similar way, RBC, Hb, and HCT were unaffected by the treatment with Ulixertinib, Ruxolitinib or their combination (Fig. S3B, D). On the contrary, the combination of Ulixertinib 75 mg/kg and Ruxolitinib 30 mg/kg led to a significant reduction of WBCs at days 8 and 14 of treatment (Fig. S3E).

As expected, splenomegaly was markedly reversed by Ruxolitinib 60 mg/kg, as revealed by the analysis of spleen volume at day 12 (Fig. 5C) and spleen index at day 15 (Fig. 5D). In addition, mice receiving Ruxolitinib at half dosage (30 mg/Kg) in combination with Ulixertinib 75 mg/kg displayed a reversion of the splenomegaly comparable with that obtained by the treatment with Ruxolitinib at full dosage (60 mg/kg) (Fig. 5C, D).

**Fig. 6:**
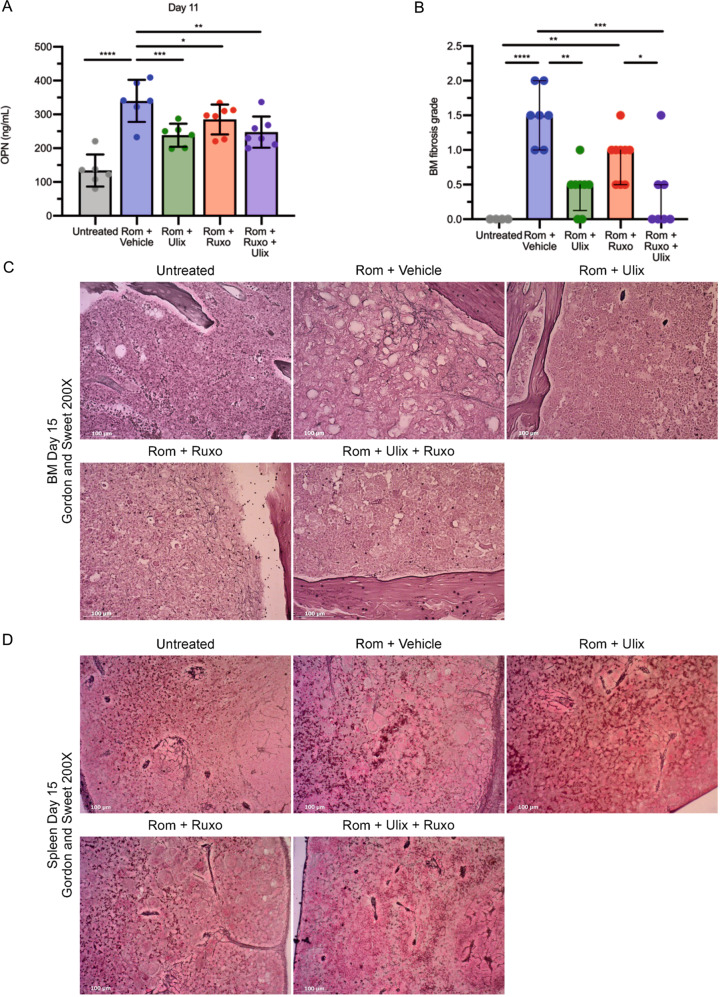
Combined ERK1/2 and JAK1/2 inhibition reduces plasma OPN and reverses BM fibrosis induced by Romiplostim. **A** OPN plasma concentration was evaluated by means of ELISA (enzyme-linked immunosorbent assay) in control mice (Untreated) and Romiplostim-treated animals receiving vehicle (Rom + Vehicle, in blue), Ulixertinib (Rom + Ulix, in green), Ruxolitinib (Rom + Ruxo, in red) or a combination of Ulixertinib and Ruxolitinib (Rom + Ruxo + Ulix, in purple). ELISA was performed 11 days after the first Romiplostim administration (*n* = 7–9/group). Histograms represent mean values while bars indicate the standard deviation. Comparisons were performed by means of one-way ANOVA. **B** Blinded fibrosis grade quantification was performed by a specialized pathologist (*n* = 4–8/group) Histograms represent median values while bars indicate the interquartile range. Comparisons were performed by means of Kruskal-Wallis’ test. **C** Representative images of Gordon and Sweet’s reticulin staining of BM sections from mice belonging to each experimental group. Magnification 200×. **D** Spleen fibrosis as shown by Gordon and Sweet’s reticulin staining. Magnification 200X. *: *P* ≤ 0.05; **: *P* ≤ 0.01; ***: *P* ≤ 0.001; ****: *P* ≤ 0.0001 Abbreviations: OPN osteopontin; Rom romiplostim; Ulix Ulixertinib; Ruxo Ruxolitinib; BM bone marrow.

Interestingly, the increase of OPN plasma levels observed upon treatment with Romiplostim was remarkably reduced by the ERK1/2 inhibition alone or in combination with JAK1/2 inhibition (Fig. 6A). Again, as demonstrated by the histological analysis of Gordon and Sweet-stained BM (Fig. 6B–C) and spleen sections (Fig. 6D), the marked decrease of plasma OPN concentration was associated with a remarkable reduction of fibrosis in mice treated with Ulixertinib alone or in combination with Ruxolitinib in comparison with mice treated with vehicle. On the other hand, the sole JAK1/2 inhibition reduced OPN plasma levels to a lower extent (Fig. 6A) and induced a slight but not statistically significant reduction of BM (Fig. 6B, C) and spleen fibrosis (Fig. 6D).

### OPN neutralization constrains the development of BM fibrosis in MF mice

**Fig. 7:**
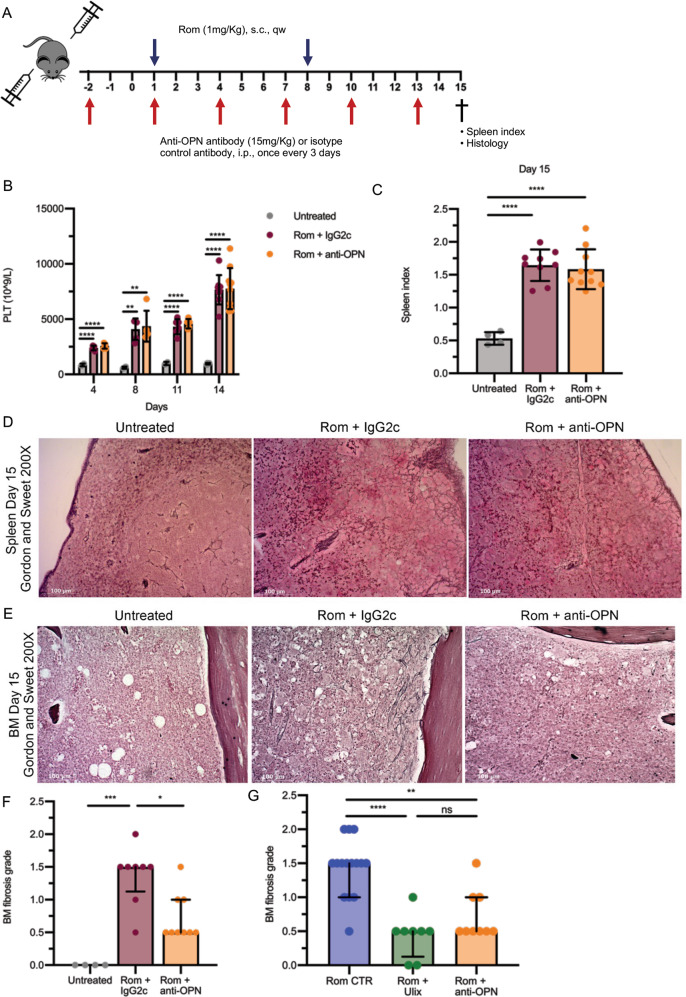
Treatment with anti-OPN neutralizing antibody recapitulates the antifibrotic effect of Ulixertinib. **A** Schematic outline of the experimental design. Myelofibrosis was induced by sub-cutaneous injection of Romiplostim (1 mg/kg, once weekly). Mice were given anti-OPN antibody 15 mg/kg (Rom + anti-OPN, in orange) or an isotype control 15 mg/kg (Rom + IgG2c, in brown) through intraperitoneal injection once every 3 days starting 3 days before the first Romiplostim injection. Mice were sacrificed after 15 days of Rom treatment for the evaluation of spleen index and bone marrow and spleen fibrosis. **B** Platelet count was assessed at days 4, 8, 11, and 14 (*n* = 4–10/group). **C** Spleen index was calculated at sacrifice (day 15) (*n* = 4–10/group). **D** Spleen fibrosis as shown by Gordon and Sweet’s reticulin staining in Untreated and treated mice. Magnification 200×. **E** Representative images of Gordon and Sweet’s reticulin staining of bone marrow (BM) sections from mice belonging to Untreated, Rom + IgG2c and Rom + anti-OPN groups. Magnification 200×. **F** Blinded fibrosis grade quantification (*n* = 4–10/group) was performed by a specialized pathologist. **G** Comparison of BM fibrosis grade reduction after ERK1/2 inhibition through Ulixertinib and anti-OPN treatment, a comprehensive analysis of data from Fig. 6B and Fig. 7F was performed (*n* = 8–15/group). In **B** and **C** histograms represent mean values while bars indicate the standard deviation; comparisons were performed by means of one-way ANOVA. In **F** and **G** histograms represent median values while bars indicate the interquartile range; comparisons were performed by means of Kruskal-Wallis’ test. *: *P* ≤ 0.05; **: *P* ≤ 0.01; ***: *P* ≤ 0.001; ****: *P* ≤ 0.0001 Abbreviations: n number of samples; s.c. sub-cutaneous; qw once weekly; i.p. intraperitoneal; OPN osteopontin; Rom romiplostim; PLT platelets; Ulix Ulixertinib; BM bone marrow.

To further clarify the role of OPN in the development of BM and spleen fibrosis we evaluated the effects of the administration of an anti-mouse OPN neutralizing antibody in Romiplostim-treated mice (Fig. 7).

Starting from 3 days before the first Romiplostim administration, an anti-OPN antibody or an isotype control IgG2c were intraperitoneally injected once every 3 days (Fig. 7A). As shown above for the pharmacological inhibition of OPN production through ERK1/2 targeting, the administration of anti-OPN antibody did not affect platelet count (Fig. 7B) and volume (Fig. S4A), as well as other hematological parameters such as WBC, RBC, Hb and HCT (Fig. S4B, E). Moreover, the development of splenomegaly was unaffected by the administration of anti-OPN antibody, as demonstrated by the evaluation of the spleen index at sacrifice (Fig. 7C).

Noteworthy, mice treated with the anti-OPN antibody showed a striking reduction of reticulin deposition in spleen (Fig. 7D) and BM (Fig. 7E, F) in comparison with mice treated with the isotype control IgG2c. Interestingly, the comparison of the qualitative (Figs. 6C and 7E) and quantitative analyses of BM fibrosis (Figs. 6B and 7F) highlighted no remarkable differences between the pharmacological inhibition of OPN production through ERK1/2 targeting and the neutralization of OPN activity by the anti-OPN antibody (Fig. 7G).

## Discussion

Primary myelofibrosis is the most aggressive among MPNs since it is characterized by an increased risk of leukemic progression and an inferior life expectancy [1]. No currently available pharmacologic approaches cure patients, since JAK1/2 inhibitors Ruxolitinib and Fedratinib are effective in reducing splenomegaly and inflammation-related symptoms but do not affect BM fibrosis and disease burden in most cases [10] and rIFN-α has proven to improve BM morphology only in a fraction of PMF patients [13, 14]. The interaction between the neoplastic clone and the surrounding microenvironment leads to the development of myelofibrosis. In particular, hematopoietic cells contribute to the development of fibrosis through the release of profibrotic molecules, like OPN, that can induce the deposition of collagen by mesenchymal stromal cells [16].In the present work we further unravel the involvement of OPN in MF pathogenesis by means of an in vivo mouse model and investigate the molecular mechanisms underlying its expression with the aim to develop novel therapeutic approaches to reduce OPN production and constrain the development of BM fibrosis.

Inhibitors of signaling pathways reported to affect SPP1 expression were evaluated in vitro in primary human monocytes given their key role in OPN production [16]. The in vitro results pointed to the ERK1/2 signaling as the most promising target since ERK1/2 inhibition by Ulixertinib successfully reduced both OPN expression and secretion. ERK1/2 pathway represents a promising therapeutic target in MF since JAK2 constitutive activation due to the presence of driver mutations results in the downstream activation of MAPK pathway together with STATs [32]. Moreover, MEK1/2 and ERK1/2 activation overcomes JAK1/2 inhibition in vivo in MPN mouse models and the administration of MEK1/2 or ERK1/2 inhibitors in combination with Ruxolitinib displayed superior therapeutic efficacy in vivo by reducing splenomegaly, disease burden and myelofibrosis [24, 25].

Among the JAK2^V617F^ knock-in mouse models [27, 33–36], the one with tissue-specific VavCRE-driven expression of JAK2V617F has been extensively used for the pre-clinic evaluation of new therapeutic strategies [27, 37] since it develops an MPN like phenotype mimicking Polycythemia Vera eventually evolving to a myelofibrotic stage in aged mice. Interestingly, we observed a remarkable increase in OPN plasma levels in JAK2^V617F^ knock-in animals at the age they develop myelofibrosis in line with results from MPN patients where OPN plasma levels were remarkably higher in PMF versus Essential Thrombocythemia and Polycythemia Vera patients, as well as in overt versus pre-fibrotic PMF patients [16]. In a similar way, OPN plasma levels resulted increased in mice treated with the MPL agonist Romiplostim. These mice develop a MF-like condition characterized by megakaryocyte hyperplasia in the BM and spleen, thrombocytosis, splenomegaly and high-grade BM and spleen fibrosis [20]. The increase in OPN plasma levels precedes the development of BM and spleen fibrosis in Romiplostim-treated mice further supporting the idea of the involvement of OPN in fibrogenesis. Since Ulixertinib should be administered twice daily [38, 39] by oral gavage -that is an invasive procedure unsustainable in the long term- we selected the Romiplostim-treated mouse model to assess in vivo the efficacy of Ulixertinib in reducing OPN production and counteract the development of myelofibrosis.

In Romiplostim-treated mice hyperplastic megakaryopoiesis leads to the development of fibrosis, while extramedullary hematopoiesis is responsible for the onset of splenomegaly [20, 40]. Our results demonstrated that ERK1/2 inhibition did not affect spleen volume which is instead reduced by JAK1/2 inhibition. On the contrary, Ulixertinib was able to significantly reduce OPN plasma concentration and the same was observed for Ruxolitinib treatment even if to a lesser extent. This is in keeping with the in vitro data we previously published [16] showing that JAK1/2 inhibition reduces OPN expression levels in PMF primary cells. Strikingly, we observed that the reduction in OPN plasma levels was always followed by a decrease in BM and spleen fibrosis. Ulixertinib significantly reduced BM and spleen fibrosis while the decrease induced by Ruxolitinib was only partial, in line with the lower effect on OPN levels. We therefore tested whether the anti-fibrotic effect of Ulixertinib was maintained in combination with Ruxolitinib. The coupled ERK1/2 and JAK1/2 inhibition efficiently reversed both fibrosis and splenomegaly even when the dosages of Ulixertinib and Ruxolitinib were reduced. Collectively, these data provide the rationale for combining ERK1/2 and JAK1/2 inhibitors to act on different pathogenic features of MF.

These results further underline the role of OPN in MF pathogenesis as a key profibrotic factor downstream of ERK1/2 signaling. In keeping with this idea, we previously demonstrated that OPN expression is positively regulated by transcription factor MAF [16] that in turn can be induced by MEK/ERK activation [41].

In order to further clarify whether the antifibrotic effect of ERK1/2 inhibition could be actually ascribed to the reduction of OPN activity, we studied the effects of the administration of anti-OPN monoclonal antibodies in Romiplostim treated mice. Strikingly, OPN neutralization resulted in a significant decrease in BM and spleen fibrosis and recapitulated the anti-fibrotic effects of ERK1/2 inhibition. Indeed, BM fibrosis grading data showed that the decrease of BM fibrosis achieved through the administration of anti-OPN antibodies was overlapping with that obtained by the Ulixertinib-mediated inhibition of OPN production.

Our results demonstrate that the anti-fibrotic effect of Ulixertinib can be ascribed to the reduction of OPN production and further emphasize the importance of OPN as a promising novel target for the treatment of MF patients.

Our data demonstrate that the interference with OPN production (through ERK1/2 inhibition) or activity (through anti-OPN antibodies) constrains the development of BM and spleen fibrosis but does not affect the extramedullary hematopoiesis -and therefore splenomegaly- in a model of constitutive activation of Mpl signaling. Our results -especially those for ERK1/2 inhibition- are in keeping with the data by Brkic demonstrating that in mice transplanted with MPLW515L-transduced BM cells ERK1/2 inhibition leads to a reduction of fibrosis without significantly affecting spleen size [24]. Mpl signaling is indeed mediated by ERK1/2, together with JAK/STAT. Our data further support the idea that the overactivation of ERK1/2 drives the abnormal OPN overproduction that is at least partially responsible for the development of BM and spleen fibrosis. On the contrary, OPN does not affect the myeloproliferation and the extramedullary hematopoiesis that leads to splenomegaly, as shown by both pharmacological inhibition and OPN neutralization.

Pre-clinical evidence for the therapeutic benefit given by the combination of JAK1/2 inhibitors and MEK or ERK inhibitors has led to phase I clinical trials aimed to evaluate the MEK inhibitor Selumetinib in combination with Azacitidine (NCT03326310) or the ERK1/2 inhibitor LTT462 in combination with Ruxolitinib (NCT04097821). Our results provide further support to the hypothesis that ERK1/2 inhibition can improve the therapeutic efficacy of Ruxolitinib, especially in terms of BM fibrosis reduction. ERK1/2 inhibitor Ulixertinib is currently under investigation for the treatment of several advanced solid tumors—such as gastrointestinal malignancies- and a phase I/II study was conducted in patients with Acute Myeloid Leukemia or Myelodisplastic Syndromes (NCT02296242) allowing the identification for the maximum tolerated dose in this cohort. Alternatively, metformin and statins, which inhibit ERK1/2 phosphorylation [42, 43], showed beneficial effects in several cancers [44, 45] and delayed myelofibrosis progression [46, 47]. Similarly, angiotensin-receptor blockers have been shown to inhibit ERK1/2 activity [48] and reduce OPN circulating levels in essential hypertension [49].

Different OPN-neutralizing monoclonal antibodies have been successfully assessed in vivo in preclinical models. For instance, AOM1 (Pfizer Inc.) is a fully human monoclonal IgG2 that cross-reacts with human and mouse OPN. The activity of AOM1 was preclinically evaluated in a mouse model of non-small cell lung cancer and demonstrated an efficient inhibition of metastatic tumor growth [50]. However, no subsequent clinical evaluation of AOM1 was performed.

Only one anti-OPN antibody, ASK8007 (Astellas Pharma Inc.), has been clinically evaluated. ASK8007 is a humanized, monoclonal antibody that blocks OPN binding to multiple cell surface receptors, such as αvβ3, αvβ5 and α_9_β_1_ integrins as well as CD44. A phase I/II clinical trial in patients with Rheumatoid Arthritis demonstrated that ASK8007 administration was well tolerated by patients with no safety concerns. However, the evaluation of co-primary efficacy endpoints highlighted a lack of clinical improvement in Rheumatoid Arthritis patients after treatment with ASK8007 [51]. The lack of clinical response could be at least partially explained by the very fast turnover rate of human OPN, with a half-life ranging between 7 and 15 min, as calculated in the serum from three heathy human subjects [52]. Pharmacokinetic and pharmacodynamic simulations suggest that sufficient target coverage by a conventional antibody would require very frequent infusions of antibody at high dosages given the very rapid OPN turnover rate [52]. Antibodies engineered to improve their pharmacokinetic, such as Sweeping Antibodies^®^, administered at high doses would allow an efficient OPN knockdown [53]. However, the feasibility of a strategy of OPN neutralization in human subjects still remains an open question.

Collectively our data univocally demonstrate the key role of OPN as an ERK1/2 downstream profibrotic effector in MF pathogenesis and point to the inhibition of OPN production by administration of Ulixertinib or the neutralization of OPN activity by anti-OPN antibodies as novel therapeutic approaches to constrain BM fibrosis development in MF patients.

## Supplementary information

Supplementary data

Supplementary Table 1

Supplementary Figure 1

Supplementary Figure 2

Supplementary Figure 3

Supplementary Figure 4

## Data Availability

Data are contained within the article or Supplementary Materials.
