# Supplementary material for: Inhibition of ERK1/2 signaling prevents bone marrow fibrosis by reducing osteopontin plasma levels in a myelofibrosis mouse model
Source: Leukemia. 2023 Mar 16;37(5):1068–79. doi: 10.1038/s41375-023-01867-3 (PMC10169646; doi:10.1038/s41375-023-01867-3)
Supplement: Supplementary file 1 — Supplementary data [file 41375_2023_1867_MOESM1_ESM.docx]

**SUPPLEMENTAL METHODS**

**Compounds and reagents**

Ulixertinib (BVD-523, cat# HY-15816) and Ruxolitinib (INCB18424, cat# HY-50856) were purchased by MedChemExpress (Monmouth Junction, NJ, USA). For *in vitro* tests, a 5 mM Ulixertinib stock solution was prepared in 100% ethanol, with final ethanol concentration ranging from 0.05% to 0.3% for *in vitro* assays. For *in vivo* studies, 100 mg/ml Ulixertinib and 250 mg/ml Ruxolitinib stock solutions were prepared in DMSO for long-term storage. Ulixertinib dosing solutions were freshly prepared by making a 1:10 dilution of the 100 mg/ml stock in a solution of SBE-β-CD 20% (w/v) in saline. Ruxolitinib dosing solutions were freshly prepared by firstly diluting the 250 mg/ml stock 1:2 in DMSO and secondly, by making a 1:10 dilution of the 125mg/ml stock in a solution of SBE-β-CD 20% (w/v) in saline. Ulixertinib 100 mg/Kg, Ruxolitinib 60 mg/Kg or a combination of Ulixertinib 75mg/Kg and Ruxolitinib 30mg/Kg were orally administered by gavage twice daily.

The thrombopoietin mimetic Romiplostim (Nplate^®^) was kindly provided by Amgen (Thousand Oaks, CA, USA). For *in vivo* studies Romiplostim was diluted in saline to reach the final concentration of 500 μg/mL and was administered by subcutaneous injection.

Anti-mouse OPN antibody (cat#BE0373, clone 103D6), the isotype control antibody (cat#BE0366, clone DV5-1) and the dilution buffer (cat#IP0070) were purchased by Bio X Cell (Lebanon, NH, USA). For *in vivo* studies, anti-mouse OPN or the isotype control antibody were diluted in the dilution buffer to reach the final concentration of 15 mg/Kg and administrated by intraperitoneal injection.

# **Isolation of human monocytes**

Human monocytes were purified upon healthy donor's informed written consent from peripheral blood samples, collected after normal deliveries, according to the institutional guidelines for discarded material and the Helsinki declaration.

Briefly, peripheral blood samples were subjected to centrifugation on a Ficoll-Hypaque gradient. Then, mononuclear cells and pellets were collected and washed twice with PBS. Monocytes were isolated by positive selection from mononuclear cells by using a magnetic cell sorting procedure (CD14 MicroBeads UltraPure, human, Miltenyi Biotec; Auburn, CA, USA). The purity of the samples was always higher than 95%, as assessed by flow-cytometry by using a PE-conjugated mouse anti-human CD14 MoAb (from Miltenyi Biotec).

**Treatment of human monocytes with Ulixertinib**

Freshly isolated CD14+ monocytes were plated at a density of 5x10^5^ cells/ml and cultured in IMDM (Euroclone, Milan, Italy) supplemented with 10% fetal bovine serum (FBS, Hyclone, Cytiva, Marlborough, MA, USA), and GM-CSF (20 ng/mL; Miltenyi Biotec; Auburn, CA, USA). After an overnight pre-activation, monocytes were treated with 0.25μM, 0.5μM, 1μM, 2.5μM, 5μM, 10μM, 15μM Ulixertinib or with the vehicle (ethanol) as control.

**Western Blot**

Monocytes were incubated with Ulixertinib or vehicle (ethanol) for 1 hour. Phospho-RSK3 and RSK-3 protein levels in monocytes were detected by Western blot analysis. Briefly: cells were harvested, washed twice with ice-cold PBS and lysed (1x10^6^ cells/20 µl of lysis buffer) in RIPA Buffer (cat. #9806, Cell Signaling Technology, Danvers, MA, USA) and protease and phosphatase inhibitors (Halt^TM^ Protease and Phosphatase Inhibitor, Cat. 78442, Thermo Scientific, Waltham, MA, USA). Total cellular lysates (20μg for each sample) were loaded onto 10% SDS-polyacrylamide gel and blotted on nitrocellulose membrane. To control loading and transfer, after transfer the membranes were stained by Red Ponceau. Membranes were then pre-blocked in blocking solution, 5% Bovine Serum Albumin in 0.1% TBST for 1 hour at room temperature (RT), incubated with primary rabbit anti-Phospho-RSK3 (1:1000 dilution overnight at 4°C; cat. #9348, Cell Signaling Technology ) or mouse anti-RSK3 (1:400 dilution overnight at 4°C; cat. SC-517283, Santa Cruz, Dallas, TX, USA) or rabbit anti-β-actin (1:5000 dilution 1hour at RT, cat. #PA1-16889, ThermoFisher Scientific, Waltham, MA, USA) antibodies. After 3 washes with TBST, blots were incubated with HRP-conjugated goat anti-rabbit secondary antibody (1:1000 dilution, cat. 32460, ThermoFisher Scientific) or goat anti-mouse secondary antibody (1:300, cat. 32430, ThermoFisher Scientific) for 1 hour at RT and revealed by Amersham^TM^ ECL Select^TM^ (cat. RPN2235, Cytiva, Marlborough, MA, USA).

**XTT viability assay**

For *in vitro* assays, CD14+ cells were seeded in 96-well culture plates with a density of 260,000 cells/well. After overnight incubation, the culture medium was replaced with fresh medium supplemented with different concentrations of Ulixertinib ranging from 2.5 to 15μM and were incubated at 37 °C under 5% CO2 for 72-96 h. TACS XTT (2,3-Bis(2-methoxy-4-nitro-5- sulfophenyl)-2H-tetrazolium-5- carbox-anilide) assay (Cat. No. 4891-025-K; R&D systems, Minneapolis, MN, USA) was performed to evaluate cell viability.

**RNA extraction**

Total cellular RNA was isolated from 3x10^5^ cells for each sample using the Qiagen miRNeasy**^®^** MicroRNA isolation kit following the manufacturer’s recommendations, as already described[1]. RNA samples concentration and purity (assessed as 260/280 nm and 260/230 nm ratios) were evaluated by NanoDrop ND-1000 spectrophotometer (NanoDrop Technologies; Wilmington, DE, USA), while RNA integrity was assessed by using the Agilent 2100 Bioanalyzer (Agilent Technologies; Waldbrunn, Germany).

**Quantitative reverse transcription polymerase chain reaction (qRT-PCR)**

Total RNA (100 ng) was reverse-transcribed to cDNA using a High Capacity cDNA Archive Kit (Life technologies; Carlsbad, CA, USA). TaqMan PCR was carried out using the TaqMan Fast Advanced PCR master mix and TaqMan gene expression assays (all reagents from Life Technologies), by means of a 7900HT Fast Real-Time PCR System (Applied Biosystems, a brand of ThermoFisher Scientific). Assays were performed in triplicate. Gene expression relative quantification was obtained through the comparative cycle threshold (CT) method, by using glyceraldehyde-3-phosphate dehydrogenase (GAPDH) as the housekeeping gene. Data were normalized by calculating ΔΔCT for each sample as the difference between its ΔCT values and the mean ΔCT value for the vehicle sample, set as calibrator; relative quantity (RQ) was expressed as 2^−ΔΔCT^.

**Enzyme-linked immunosorbent assay (ELISA)**

The levels of OPN in (a) culture supernatant from human monocytes *in vitro* treated with Ulixertinib and (b) plasma samples from Romiplostim-treated mice treated with Ulixertinib or vehicle were analyzed by ELISA by using human OPN (cat#SOST00) or mouse/rat OPN (cat#MOST00) Quantikine kits, respectively (both from R&D Systems, Minneapolis, MN) according to the manufacturer's instructions. Each sample was assayed in duplicate.

**Tissue specimen processing and histological analyses**

Formalin-fixed femurs were decalcified in acidic solution containing EDTA (Osteodec, cat#05-M03005, Bio-Optica, Milan, Italy) for 4 days at room temperature. Spleen and bones were paraffin-embedded. Next, 4-μm tissue sections were cut by using a Leica RM2235 (Leica biosystems, Deer Park, IL, USA) and processed for Gordon and Sweet's Silver Staining for reticulin detection (cat#04-040802, Bio-Optica, Milan, Italy).

Quantitation of BM fibrosis was performed by a pathologist in keeping with the WHO grading criteria [2] integrated with the addition of intermediate levels of fibrosis grading (MF-0.5 and MF-1.5) [3–5] as detailed in **Table 1**.

The images were captured by using an Axioscope A1 microscope equipped with an AxioCam ERc 5S Digital Camera and Axion software 4.8 (all Carl Zeiss MicroImaging Inc.; Thornwood, NY, USA).

**REFERENCES**

1 . Bianchi E, Bulgarelli J, Ruberti S, Rontauroli S, Sacchi G, Norfo R et al. MYB controls erythroid versus megakaryocyte lineage fate decision through the miR-486-3p-mediated downregulation of MAF. Cell Death Differ 2015; 22: 1906–1921.

2 . J Thiele, HM Kvasnicka, F Facchetti, V Franco, J van der Walt, A Orazi. European consensus on grading bone marrow fibrosis and assessment of cellularity. Haematologica 2005; 90: 1128–1132.

3 . Brkic S, Stivala S, Santopolo A, Szybinski J, Jungius S, Passweg JR et al. Dual targeting of JAK2 and ERK interferes with the myeloproliferative neoplasm clone and enhances therapeutic efficacy. Leukemia 2021; 35: 2875–2884.

4 . Stivala S, Codilupi T, Brkic S, Baerenwaldt A, Ghosh N, Hao-Shen H et al. Targeting compensatory MEK/ERK activation increases JAK inhibitor efficacy in myeloproliferative neoplasms. J Clin Invest 2019; 129: 1596–1611.

5 . Leimkühler NB, Gleitz HFE, Ronghui L, Snoeren IAM, Fuchs SNR, Nagai JS et al. Heterogeneous bone-marrow stromal progenitors drive myelofibrosis via a druggable alarmin axis. Cell Stem Cell 2021; 28: 637-652.e8.

**SUPPLEMENTAL FIGURE LEGENDS**

**Supplementary Figure 1: ERK1/2 inhibition does not affect blood count in mice treated with Romiplostim.** Results of blood count obtained using Heska HT5 hematology analyser. Mean platelet volume (MPV) (**A**), white blood cell count (WBC) (**B**), red blood cell count (RBC) (**C**), hemoglobin (Hgb) (**D**) and hematocrit (HCT) (**E**) were assessed at days 4, 8, 11 and 14 (n=4-9/group). Mice treated with Romiplostim (Rom) alone (Rom + Vehicle, in blue) or with Rom and Ulixertinib (Rom + Ulix, in green) were compared with untreated animals (grey). Histograms represent mean values while bars indicate the standard deviation. Comparisons were performed by means of one-way ANOVA test. *: P≤0.05; **: P≤0.01; ***: P≤0.001. Abbreviations: n: number of samples; MPV: mean platelet volume; WBC: white blood cells; RBC: red blood cells; Hgb: hemoglobin; HCT: hematocrit; Rom: Romiplostim; Ulix: ulixertinib.

**Supplementary Figure 2: Spleen volume calculated through ultrasound ecography correlates with spleen index. A)** Representative images of mouse spleens treated and untreated with Romiplostim (Rom). For each spleen, the image shows the maximum longitudinal area. **B)** Representative images of spleens harvested at sacrifice from mice treated or untreated with Rom. **C)** After spleen monitoring by ultrasounds, scans were converted in a 3D model using Vevo Lab 3.1.0 software and spleen volume was quantified. **D)** Representative images of the 3D spatial structure of a mouse spleen (in light blue) analyzed by ultrasounds. **E)** Scatter plot representing the correlation between spleen volume and spleen index. The spleen index was calculated at sacrifice within 24 hours from the ecographic evaluation of the spleen volume. The Spearman correlation was computed to measure and identify the degree of linear dependence between the ranked variables. Each dot represents a mouse.

**Supplementary Figure 3: Combined ERK1/2 and JAK1/2 inhibition reduces white blood cells count in Romiplostim-treated mice.** Results of blood count obtained using Heska HT5 hematology analyser. Mean platelet volume (MPV) (**A**), white blood cell count (WBC) (**B**), red blood cell count (RBC) (**C**), hemoglobin (Hb) (**D**) and hematocrit (HCT) (**E**) were assessed at days 4, 8, 11 and 14 (n=4-9/group). Histograms represent mean values while bars indicate the standard deviation. Comparisons were performed by means of one-way ANOVA test. *: P≤0.05; **: P≤0.01; ***: P≤0.001. Abbreviations: n: number of samples; MPV: mean platelet volume; WBC: white blood cells; RBC: red blood cells; Hb: hemoglobin; HCT: hematocrit; Rom: Romiplostim; Ulix: ulixertinib; Ruxo: ruxolitinib.

**Supplementary Figure 4: OPN inhibition does not affect blood count in mice treated with Romiplostim.** Results of blood count obtained using Heska HT5 hematology analyser. Mean platelet volume (MPV) (**A**), white blood cell count (WBC) (**B**), red blood cell count (RBC) (**C**), hemoglobin (Hb) (**D**) and hematocrit (HCT) (**E**) were assessed at days 4, 8, 11 and 14 (n=4-9/group). Histograms represent mean values while bars indicate the standard deviation. Comparisons were performed by means of Kruskal-Wallis’ test. *: P≤0.05; **: P≤0.01; ***: P≤0.001. Abbreviations: n: number of samples; MPV: mean platelet volume; WBC: white blood cells; RBC: red blood cells; Hgb: hemoglobin; HCT: hematocrit; Rom: Romiplostim; OPN: osteopontin.
