# Supplementary figures and images for: Inhibition of ERK1/2 signaling prevents bone marrow fibrosis by reducing osteopontin plasma levels in a myelofibrosis mouse model
Source: Leukemia. 2023 Mar 16;37(5):1068–79. doi: 10.1038/s41375-023-01867-3 (PMC10169646; doi:10.1038/s41375-023-01867-3)

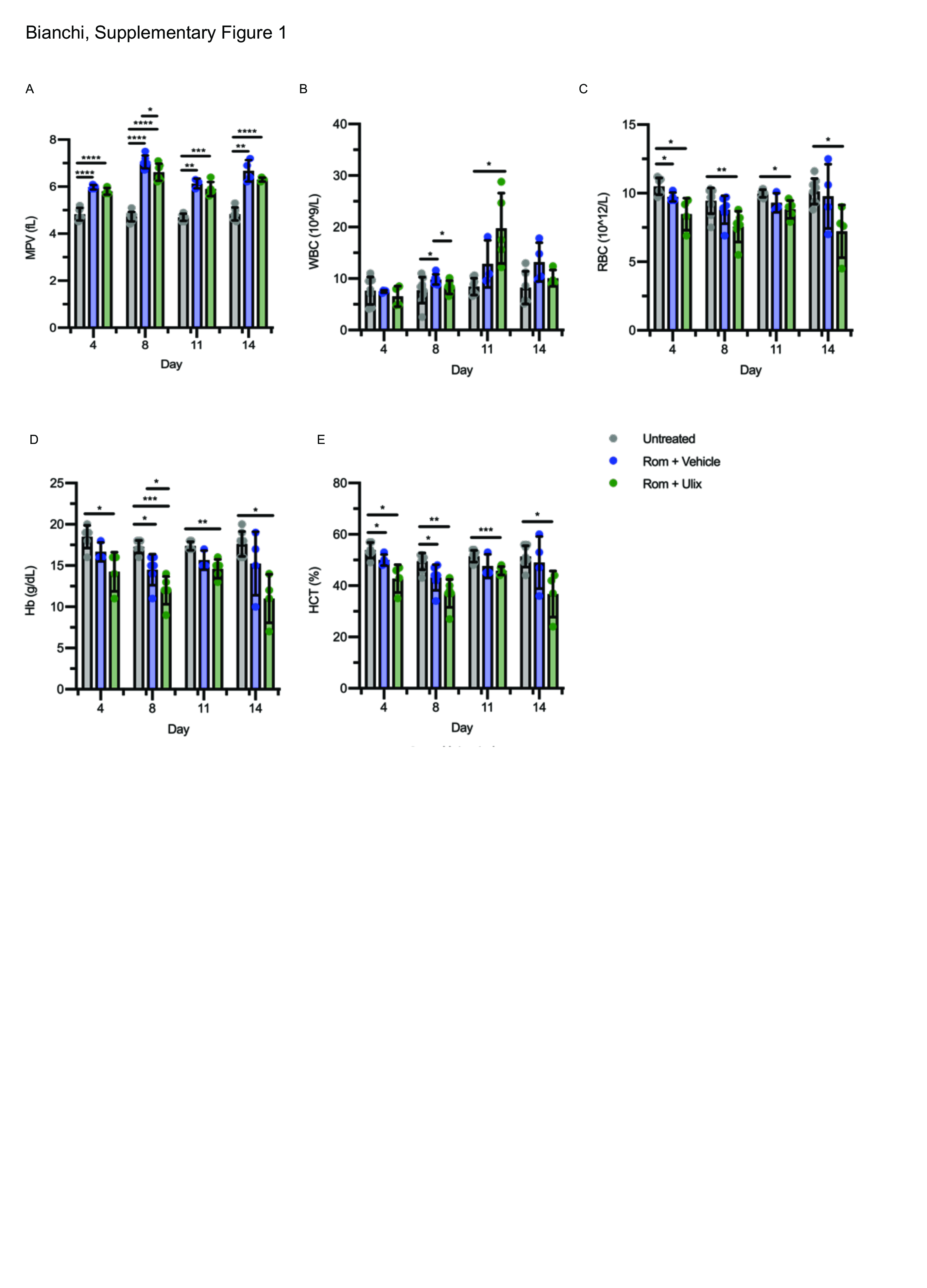

Supplement: Supplementary file 3 — Supplementary Figure 1 [file 41375_2023_1867_MOESM3_ESM.tif]

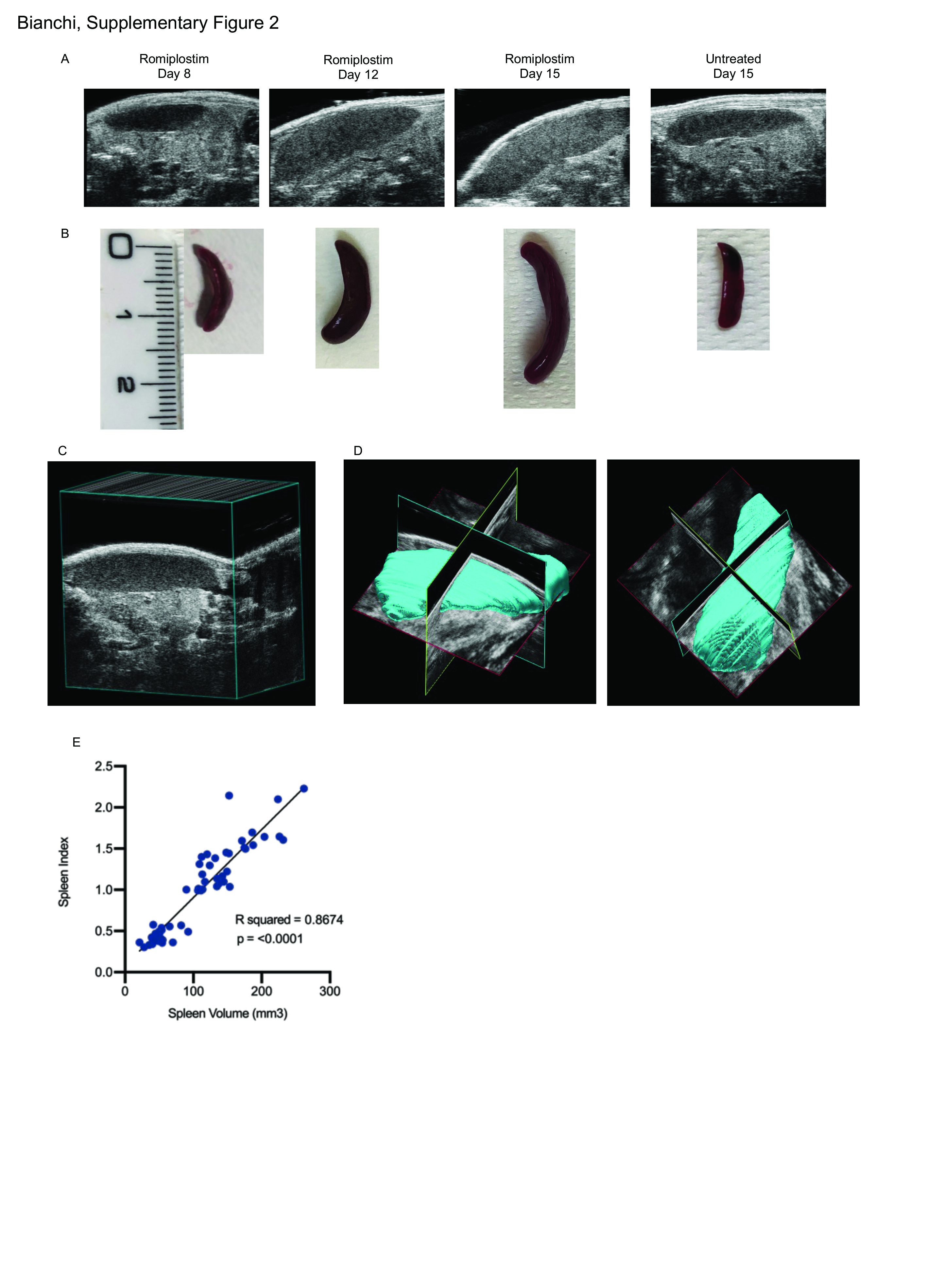

Supplement: Supplementary file 4 — Supplementary Figure 2 [file 41375_2023_1867_MOESM4_ESM.tif]

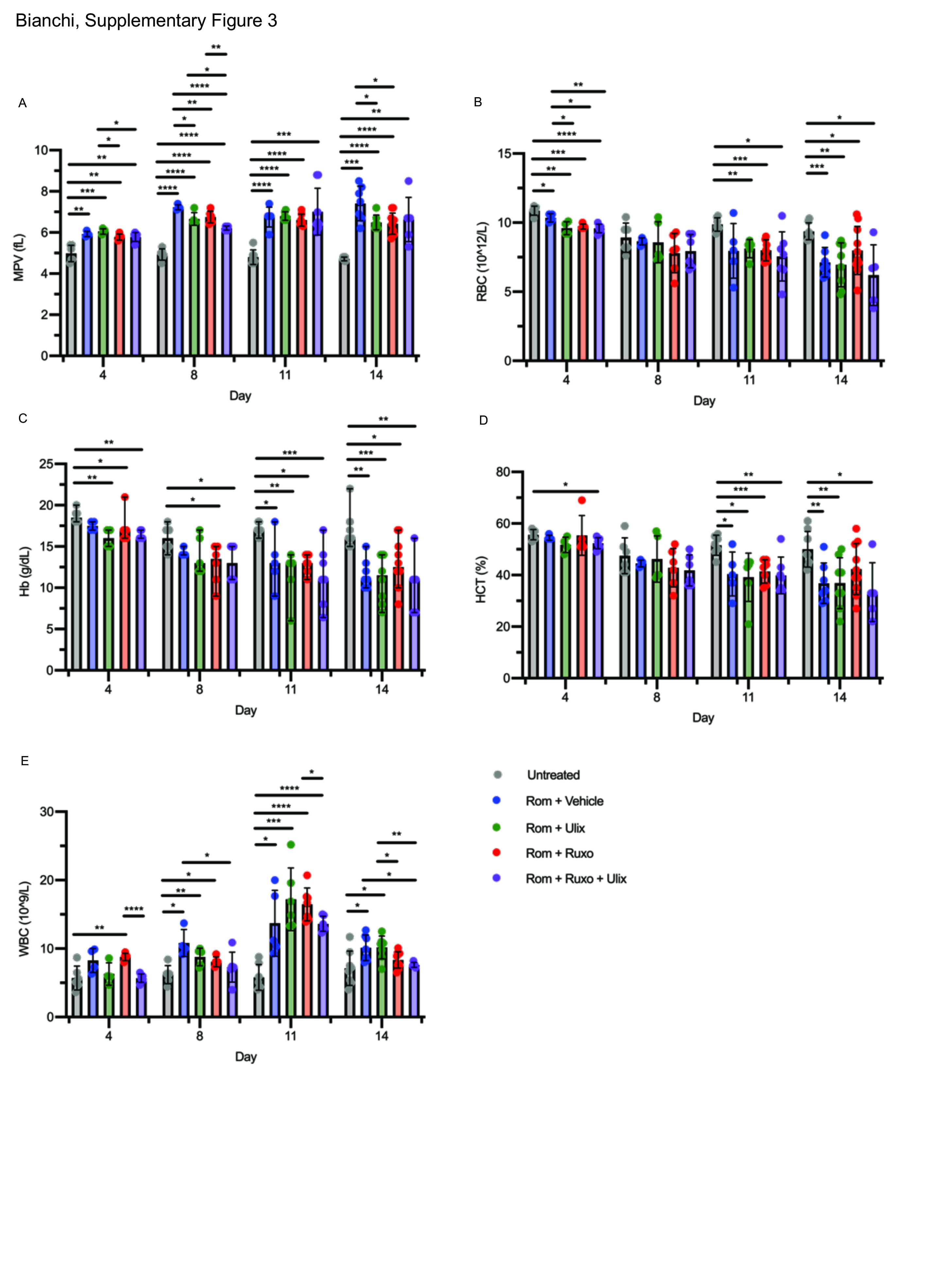

Supplement: Supplementary file 5 — Supplementary Figure 3 [file 41375_2023_1867_MOESM5_ESM.tif]

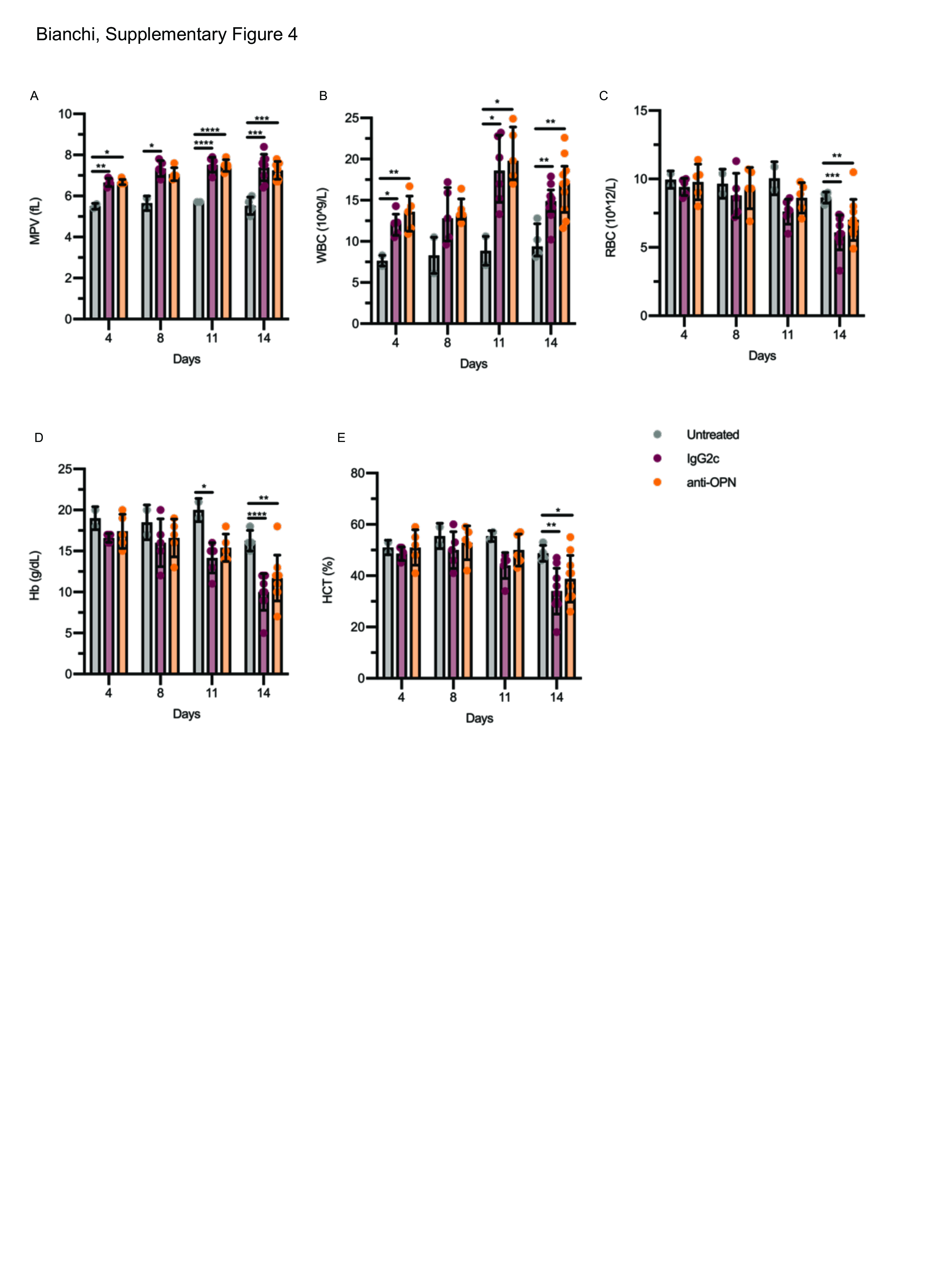

Supplement: Supplementary file 6 — Supplementary Figure 4 [file 41375_2023_1867_MOESM6_ESM.tif]
